# Urolithin C suppresses colorectal cancer progression via the AKT/mTOR pathway

**DOI:** 10.1007/s11418-024-01821-2

**Published:** 2024-06-07

**Authors:** Haochi Yang, Binghuo Wu, Qi yang, Tian Tan, Dan Shang, Jie Chen, Chenhui Cao, Chuan Xu

**Affiliations:** 1 School of Medical and Life Sciences, Chengdu University of Traditional Chinese Medicine, Chengdu, 611137 China; 2 Department of Oncology and Cancer Institute, Sichuan Academy of Medical Sciences, Sichuan Provincial People’s Hospital, University of Electronic Science and Technology of China, Chengdu, 610072 China; 3 Department of Laboratory Medicine, Sichuan Provincial Key Laboratory for Human Disease Gene Study, Sichuan Provincial People’s Hospital, University of Electronic Science and Technology of China, Chengdu, 610072 China; 4 Biotherapy Centre, Harbin Medical University Cancer Hospital, Harbin, 150081 China; 5 School of Medicine, University of Electronic Science and Technology of China, Chengdu, 610047 China; 6 Sichuan Cancer Hospital and Institute, Sichuan Cancer Centre, School of Medicine, University of Electronic Science and Technology of China, Chengdu, 610042 China; 7 Yu-Yue Pathology Scientific Research Centre, Chongqing, 400039 China; 8 Jinfeng Laboratory, Chongqing, 401329 China

**Keywords:** Urolithin C, Colorectal cancer progression, YBX1, AKT/mTOR pathway

## Abstract

Urolithin families are gut-microbial metabolites of ellagic acid (EA). Although urolithin A (UA) and urolithin B (UB) were reported to have antiproliferative activities in cancer cells, the role and related mechanisms of urolithin C (UC) in colorectal cancer (CRC) have not yet been clarified. In this study, we assess the antitumor activities of UC in vitro and in vivo and further explore the underlying mechanisms in CRC cell lines. We found that UC inhibited the proliferation and migration of CRC cells, induced apoptosis, and arrested the cell cycle at the G2/M phase in vitro, and UC inhibited tumor growth in a subcutaneous transplantation tumor model in vivo. Mechanically, UC blocked the activation of the AKT/mTOR signaling pathway by decreasing the expression of Y-box binding protein 1(YBX1). The AKT agonist SC79 could reverse the suppression of cell proliferation in UC-treated CRC cells. In conclusion, our research revealed that UC could prevent the progression of CRC by blocking AKT/mTOR signaling, suggesting that it may have potential therapeutic values.

**Graphical Abstract:**

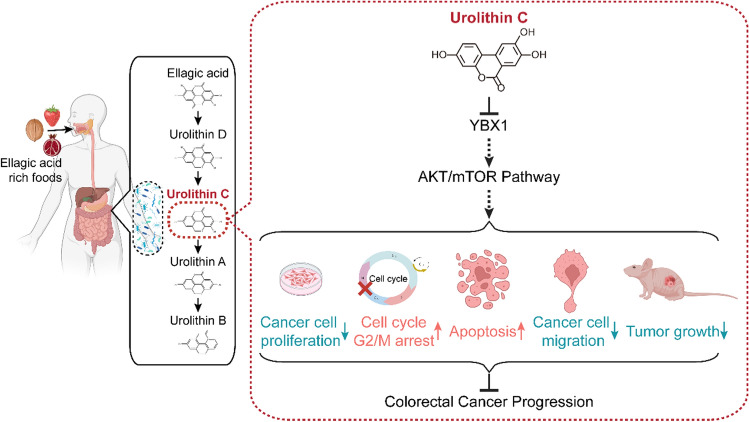

**Supplementary Information:**

The online version contains supplementary material available at 10.1007/s11418-024-01821-2.

## Introduction

Colorectal cancer (CRC) is the third-most common cancer in the world [1]. Traditional treatment for CRC includes surgery, chemotherapy, radiotherapy, and targeted therapy [2, 3]. Unfortunately, most CRC patients are diagnosed at an advanced stage, which means they are no longer eligible for surgery [4]. CRC exhibits a high susceptibility to drug resistance and metastatic recurrence, leading to a poor prognosis and an exceptionally low 5 year survival rate [5]. Therefore, there is an urgent need to explore the molecular mechanisms of tumor progression, and the search for drugs that target these processes may have potential clinical and social implications.

Recently, natural compounds have generated widespread attention in the prevention and treatment of tumors [6]. In China, several natural compound preparations, including icariin, huabanin, and zanzibaru, have been applied in clinics and have demonstrated remarkable efficacy in the treatment of tumors [7–9]. Urolithins are the metabolite of ellagic acid (EA). With the presence of gut microbes, EA is metabolized to urolithins by loss of a lactone ring and gradual dihydroxylation [10]. Urolithins, belonging to the benzo coumarin group, are recognized by their chemical structure, which is based on the amount of hydroxyl groups [11]. Numerous studies have indicated that urolithins play an important part in the activation of antioxidative, antitumor, anti-inflammatory, and mitochondrial autophagy induction [10, 12]. Among them, urolithin A (UA) has garnered significant attention due to its remarkable anticancer activity against bladder, hepatocellular, lung, prostate, colorectal, and pancreatic cancers [13–20]. Besides, numerous researches have demonstrated that urolithin B (UB) has an anticancer impact on colorectal, liver, and breast cancers [20–22].

Some studies have shown that urolithin C (UC) has antitumor effects in prostate cancer cells, bladder cancer cells, and colorectal cancer [22–24]. It is interesting to note that most researches of UC focus on its antidiabetic effect, antiobesity effect, anti-inflammatory effect, and anticancer effect. Research has shown that UC can activate the intracellular ERK1/2 signaling and Nrf2 signaling pathway, which can affect β-cell function and increase insulin release [25, 26]. Besides, UC functions as a glucose-dependent activator of insulin secretion acting by promoting Ca^2 +^ influx into pancreatic β-cells and the opening of *L*-type Ca^2 + ^channels [27]. Another study has shown that UC has a role in both the initiation and progression of inflammatory diseases by specifically regulating neutrophil activity [28]. Research has demonstrated that UC can prevent obesity by enhancing fatty acid oxidation and reducing triglyceride accumulation in adipocyte hepatocytes [29]. However, the biological role and underlying mechanism by which UC inhibits colorectal cancer are still unknown.

In the present study, UC inhibited cell proliferation, promoted apoptosis, suppressed the cell cycle at the G2/M phase, and also inhibited the migration of CRC cells. Moreover, UC significantly suppressed tumor growth in a transplantation tumor model. Mechanically, our results demonstrated that UC inhibited the expression of *Y*-box binding protein 1 (YBX1) and consequently inhibited its key downstream AKT/mTOR signaling pathway. Overall, our study reveals that UC has antitumor effects both in vitro and in vivo, which may provide a potential approach for the treatment of CRC.

## Materials and methods

### Regents and antibodies

UC (TN7108, Topscience, Shanghai, China) and SC79 (T2274, Topscience, Shanghai, China) were dissolved in dimethyl sulfoxide (DMSO) (67-68-5, MP Biomedicals, Santa Ana, USA). The antibodies were listed as follows: rabbit anti‐GAPDH (10,494-1-AP, Proteintch, Wuhan, China), rabbit anti‐AKT (pan) (C67E7) (4691S, CST, Boston, USA), rabbit anti‐phospho-Akt (Ser473) (4060S, CST, Boston, USA), rabbit anti‐mTOR (7C10) (2983S, CST, Boston, USA), rabbit anti‐phospho-mTOR (Ser2448) (2971S, CST, Boston, USA), rabbit anti‐YBX1 (HY-P80936, MCE, State of New Jersey, USA), and the secondary antibody HRP-conjugated Affinipure Goat Anti-Rabbit IgG(H + L) (HY-P80936, Proteintech, Wuhan, China) for Western blot (WB).

### Cell culture

All cell lines (CRC cell lines DLD1, HCT116 and RKO and the HEK293T cell lines) were purchased from the American type culture collection ATCC (Manassas, VA, USA). And all cells were cultured in RPMI-1640 (C22400500CP, Gibco, Waltham, MA, USA) and 10% fetal bovine serum (FBS) (F101-01, Vazyme, Nanjing, China) in an incubator with 5% CO_2_ at 37 °C.

### Cell viability assay

A total of 4000 CRC cells were seeded in a 96-well plate and incubated overnight. CRC cells were subjected to treatment with dimethyl sulfoxide (DMSO) at a concentration of 0.1% as the control, along with varying concentrations of UC (12.5, 25, 50, 100, and 200 μM) for 24, 48, and 72 h. Cell viability was assessed using the Cell Counting Kit-8 (CCK-8) assay (C0005, Topscience, Shanghai, China) following the provided procedure. The absorbance at 450 nm was measured after a 2 h incubation at 37 °C using a microplate reader.

### Colony formation assay

CRC cells were plated in six‐well plates and contained 2000 cells per well. After incubation overnight, these were treated with UC (15, 30 µM) for another 72 h. The medium was changed every 3 days. 14 days later, 4% paraformaldehyde (BL539A, Biosharp, Anhui, China) was used for fixing cells for 25 min and staining with 0.1% purple crystal (G1062, Solarbio, Beijing, China) for 25 min. Photographs were taken and the cell colonies were manually counted.

### Apoptosis assays and cell cycle analysis

The FITC–Annexin V Apoptosis Detection Kit (A211-01, Vazyme, Nanjing, China) was used to detect apoptosis. CRC cells were harvested after incubation with 15 and 30 µM UC for 72 h and washed in cold PBS twice. Staining was performed using 5 µL of FITC–annexin V and 5 µL of propidium iodide. After 25 min of incubation at room temperature (25 °C) and protection from light, 400 µL of 1X binding buffer was added, then analyses were performed within 1 h using a flow cytometer (FCM) (BECKMAN COULTER, California, USA). The Cell Cycle Kit (F10797 Invitrogen, California, USA) was used to conduct the cell cycle analysis. CRC cells were harvested after incubation with 15 and 30 µM UC for 72 h and fixed in 70% ethanol overnight. Cells were stained with 500 µL FxCycle™ PI/RNase Staining Solution for 15–30 min at room temperature and protected from light. Then the FCM (BECKMAN COULTER, California, USA) was used to analyze PI-positive cells according to the given protocol.

### Wound-healing assay

A sterile 200µl tip was used to create a straight wound for analysis. Cells were incubated with UC (15, 30 µM) in a six-well plate, and the straight wounds were photographed and measured under a microscope at 0, 24, and 48 h.

### Transwell assay

1 × 10^5^ (DLD1 and RKO) cells were cultured in the top chamber of the 24-well plate with serum-free FBS and incubated with UC (15, 30 µM). The bottom chamber was cultured in RPMI-1640 medium with 30% FBS [30]. 4% paraformaldehyde and 0.1% purple crystal were used to fix and stain the invading cells after incubation for 24 h, and then a microscope was used to count the cells.

### Mouse xenograft model

To create the xenograft model, 5 × 10^6^ DLD1 cells were injected into the flank of nude mice purchased from Beijing Huafukang Bio-Tech Co. Once the tumor volume reached 20 mm^3^, the mice were randomly divided into the control group and UC group (*n* = 5 per group). The mice received UC (intraperitoneal injection, 5 mg/kg, 3 times a week) or DMSO according to the indicated assay. Tumor volume was measured and calculated every 2 days, volume = length × width^2^/2. The tumors were harvested and weighed after 3 weeks. Paraformaldehyde was used to fix the tumor for H&E staining (HE). The Ethics Committee of Animal Experiments of Sichuan Provincial People’s Hospital approved all animal experiments.

### H&E staining

Collected tissues were fixed and preserved by 4% paraformaldehyde at 4℃ for 72 h [31]. Then the tissue samples were sequentially fixed, dehydrated, embedded, and sectioned. Then, the sections were stained with the H&E staining kit (BL700A, Biosharp, Anhui, China) according to the manufacturer’s instructions.

### RNA‐sequencing analysis

Trizol reagent (R411-01, Vazyme, Nanjing, China) was used to extract RNA in accordance with the protocol after treatment with DMSO and UC (15 µM) for 72 h. RNA was sequentially tested for quality, integrity, and quantity. Then the samples were submitted to sequencing (HaploX Biotech, Shenzhen, China). We screened the differential transcription factor genes among samples for Foldchange and Padj corresponding to the up- and downregulated transcription factor genes with the set threshold: | log2 (Foldchange) |> 1 and Padj < 0.05. The downregulated genes are in blue and upregulated genes in red.

### Quantitative reverse transcription PCR (RT-qPCR)

The RNA extraction kit (RC112, Vazyme, Nanjing, China) and reverse transcription reagent (R302-01, Vazyme, Nanjing, China) were used to extract the RNA and reverse transcription of the aimed cDNA. The SYBR qPCR reagents (Q712, Vazyme, Nanjing, China) were used for processing qPCR experiment according to the manufacturer’s instructions. We used the 2^−∆∆Ct^ method to calculate the relative expression levels of each gene. The primer sequences are presented in Table 1.

**Table 1:** Primer sequences for RT-qPCR

Gene name	Primers	Sequence (5‵ → 3‵)
YBX1	Forward	TAGACGCTATCCACGTCGTAG
	Reverse	ATCCCTCGTTCTTTTCCCCAC
GAPDH	Forward	CTGGGCTACACTGAGCACC
	Reverse	AAGTGGTCGTTGAGGGCAATG

### Lentiviral transduction assays

Lentiviral vectors with YBX1 and control sequences were designed by Geneppl (Geneppl technology Co. Nanjing, China) and transductions were conducted using the manufacturer’s protocol.

### Western blot

Cells were lysed on ice for 15 min in RIPA cell lysis buffer (P70100, NCM, Suzhou, China) and phosSTOP phosphatase inhibitor (P002, NCM, Suzhou, China). The protein lysate was centrifuged and the upper clearance collected. Using the BCA Protein Assay Kit (PD101-250 T, Oriscience, Chengdu, China) to measure the concentration of the protein, the protein was separated on SDS–polyacrylamide gels and transferred to polyvinylidene difluoride membranes (10,600,023, Cytiva, Marlborough, MA, USA). After incubation overnight at 4 °C with the primary antibody and incubating with the secondary antibody for 1 h at room temperature, the protein bands were detected and visualized on Mini Chemiluminescent/Fluorescent Imaging and Analysis System (Sinsage, Beijing, China).

### Statistical analysis

GraphPad Prism 9 was used to process the data. The Student’s *t* test was used for the two groups’ comparison. ANOVA was used for multiple comparisons between more than two groups. All experiments were presented three times independently, and the results were considered significant at *p* < 0.05 (*p < 0.05, **p < 0.01, ***p < 0.001).

## Results

### UC inhibits cell proliferation in CRC cells

**Fig. 1:**
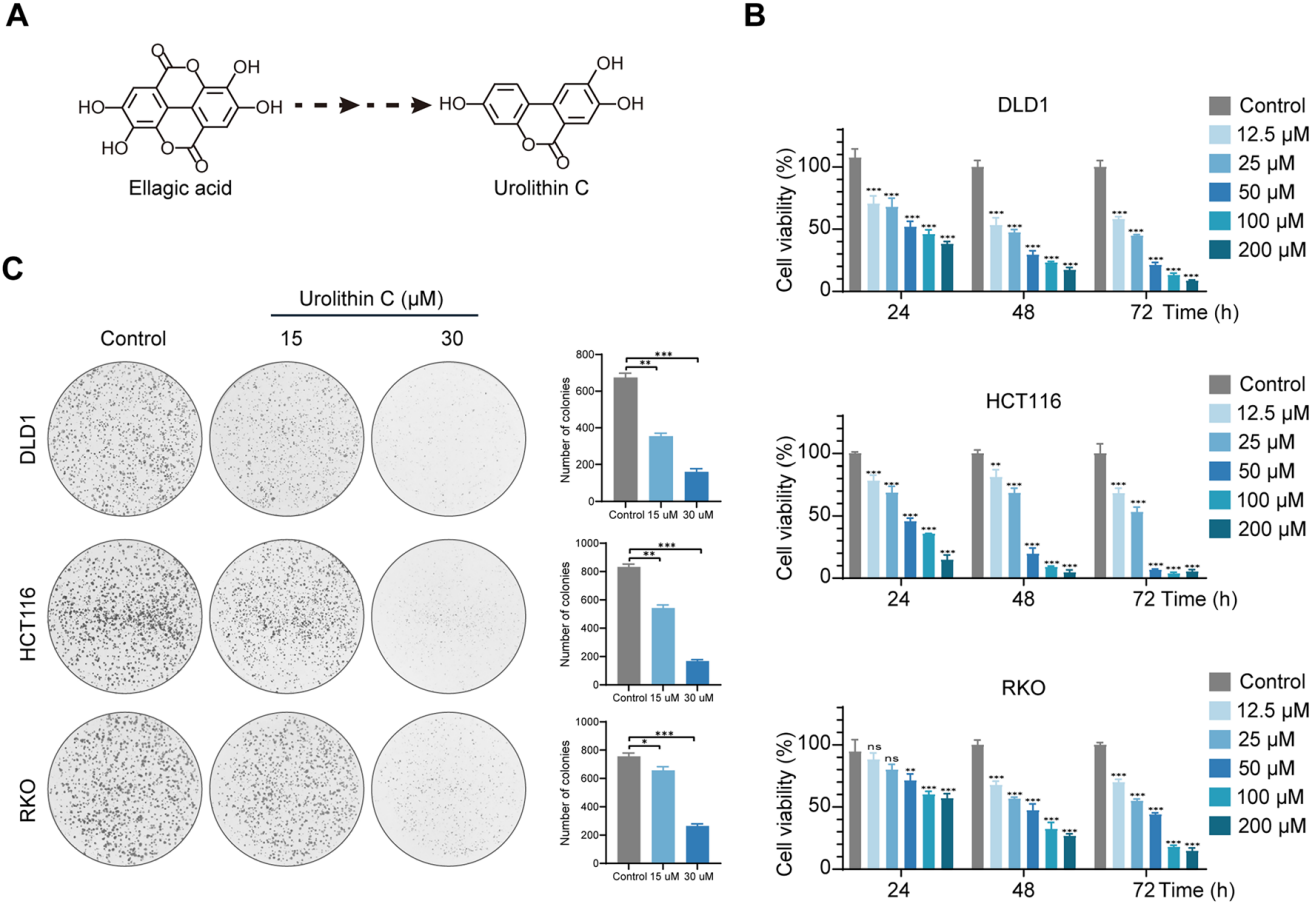
UC inhibits CRC cells proliferation. **A** UC was synthesized from EA through a variety of metabolic activities. **B** CRC cells were treated with serial concentrations of UC for 24, 48 h, and 72 h. The effects of UC on cell proliferation were measured by CCK8 assays, and cell viability was examined (*n* = 3). **C** CRC cells were treated with UC for 72 h, maintained in the medium for another 14 days, and the colony formation was analyzed (*n* = 3). The results are shown as mean ± SD, ns, not significant, *P < 0.05, **P < 0.01, ***P < 0.001 in two-way ANOVA (**B**) or in one-way ANOVA (**C**)

UC was synthesized from EA through a variety of metabolic activities (Fig. 1A). To determine whether UC inhibits the growth of CRC cells, CRC cells were treated with DMSO and UC (12, 5, 25, 50, 100 and 200 µM) for 24, 48, and 72 h, respectively. The result demonstrated that UC suppresses the proliferation of CRC cells in a dose- and time-dependent manner (Fig. 1B) and the 50% inhibitory concentration (IC50) of RKO, HCT116, and DLD1 cells were 28.81 μM, 23.06 μM, and 14.7 μM when treated with UC for 72 h. Meanwhile, CRC cells were treated with DMSO and UC (15, 30 µM) and the results showed a dramatic reduction in colony formation numbers after UC treatment in a dose‐dependent manner (Fig. 1C). Overall, the above results revealed that UC could inhibit CRC cell proliferation.

### UC induces apoptosis and cell cycle arrest in CRC cells

**Fig. 2:**
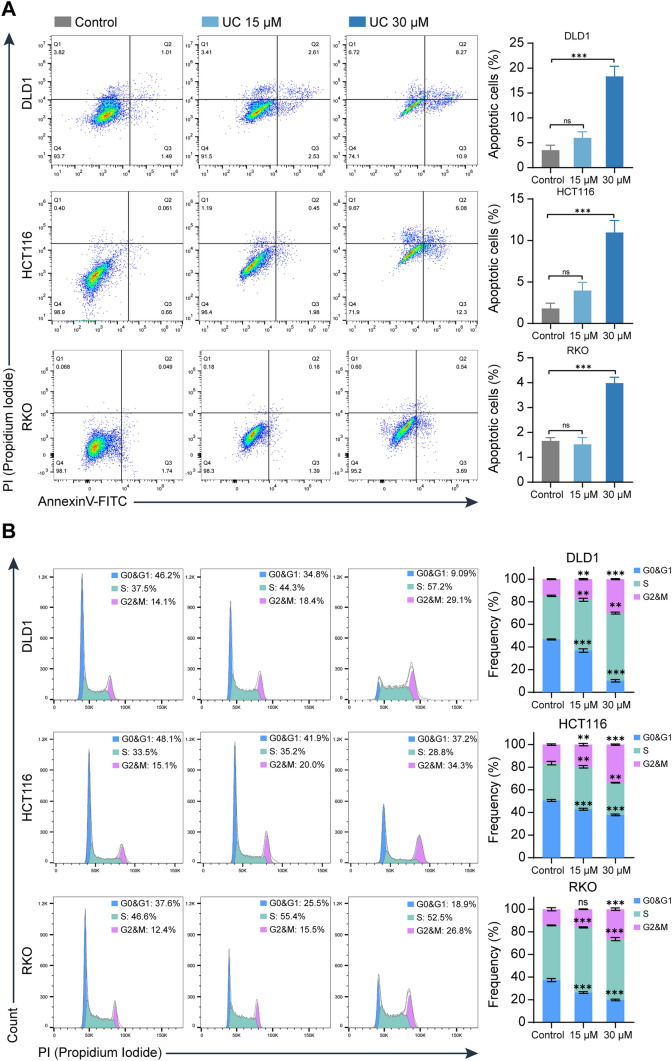
UC induces apoptosis and cell cycle arrest in CRC cells. **A** CRC cells were treated with UC (15 μM and 30 μM) for 72 h, and the apoptotic cells were examined by annexin V–FITC and PI double staining (*n* = 3). **B** CRC cells were treated with UC (15 μM and 30 μM) for 72 h, and cell cycles were determined by FCM (*n* = 3). Data are presented as mean ± SD, ns, not significant, **P < 0.01, ***P < 0.001 in one-way ANOVA (**A**) or in two-way ANOVA (**B**)

We further investigated the UC-treated CRC cells for apoptosis detection and cell cycle analysis. The result showed that there is no difference between control and UC (15 µM) in the percentage of apoptotic cells. When CRC cells were treated with UC (30 µM) (Fig. 2A), the percentage of apoptotic cells was increased. As shown in Fig. 2B, DLD1 and HCT116 cells are arrested at the G2/M phase compared to the control in a dose-dependent manner after CRC cells were treated with UC for 72 h (Fig. 2B). After UC (15 µM) treatment of RKO cells, there was no difference in cell cycle compared to the controls. However, when UC was treated with the concentration of 30 µM, RKO cells obviously underwent arrest at the G2/M phase (Fig. 2B). Above all, UC may induce apoptosis and cell cycle G2/M phase arrest in CRC cells.

### UC inhibits the migration of CRC cells

**Fig. 3:**
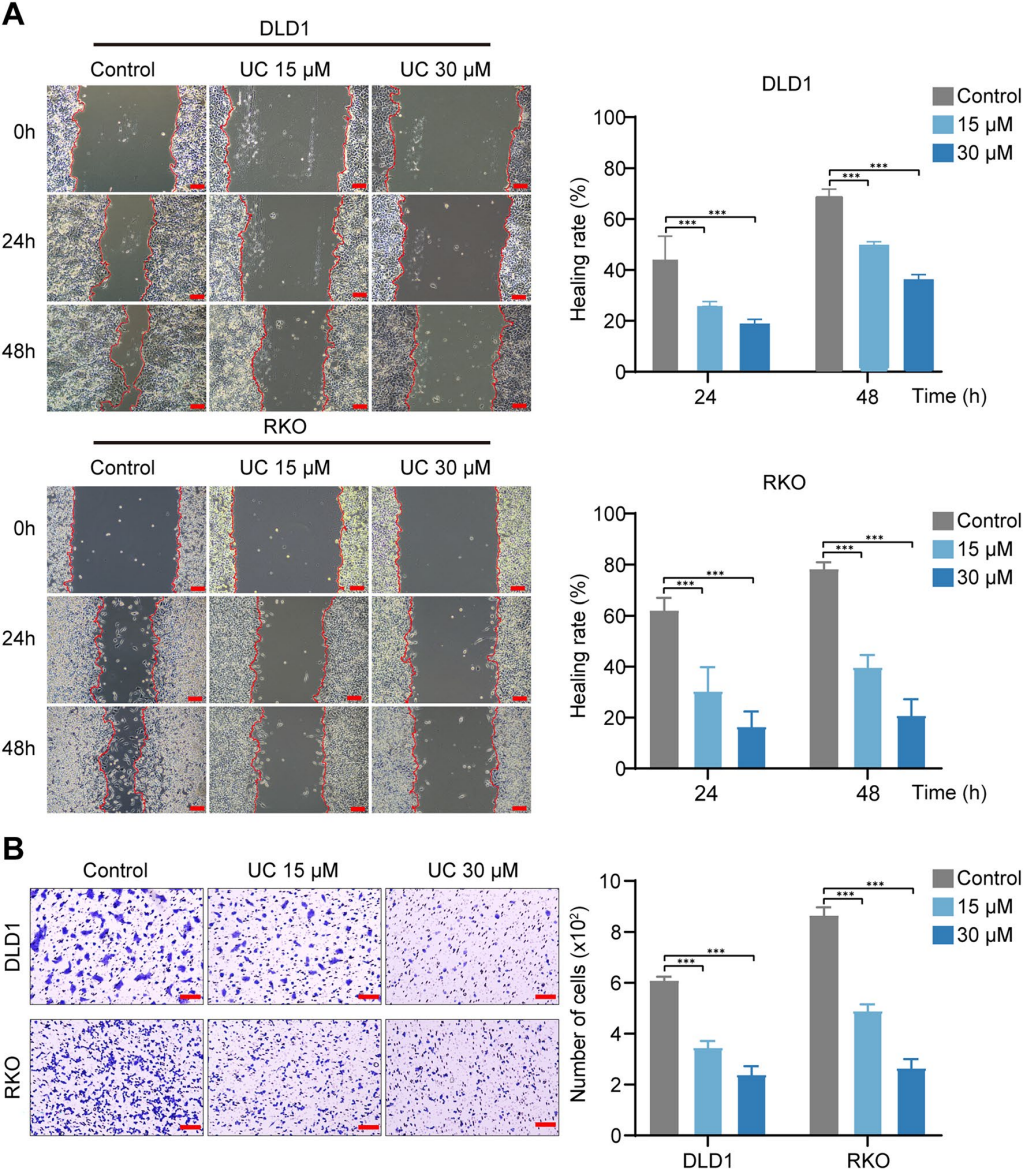
UC inhibits the migration of CRC cells. **A** Wound healing assay was conducted to assess the effect of UC (15 μM and 30 μM) on the migration of DLD1 and RKO cells 24 h and 48 h after treatment (*n* = 3), Scale bar = 100 μm. **B** Migration of DLD1 and RKO cells treated with UC (15 μM and 30 μM) was assessed by transwell assays (*n* = 3), Scale bar = 100 µm. Data were presented as mean ± SD, ***P < 0.001 in two-way ANOVA (***A***, ***B***)

A previous study has shown the role of urolithins on tumor migration [32], so we also investigated the effect of migration on UC-treated CRC cells. Next, wound healing assays and transwell assays were performed to validate the effect of UC on cell migration in CRC cells. The migration of CRC cells was greatly reduced in a dose-dependent manner with the treatment of UC (Fig. 3A). Besides, transwell experiments show that the invaded cells were greatly decreased in CRC cells when treated with UC (Fig. 3B), especially in the concentration of 30 µM. Overall, the result revealed that UC could effectively inhibit the migration of CRC cells.

### *UC suppresses xenograft tumor growth *in vivo

**Fig. 4:**
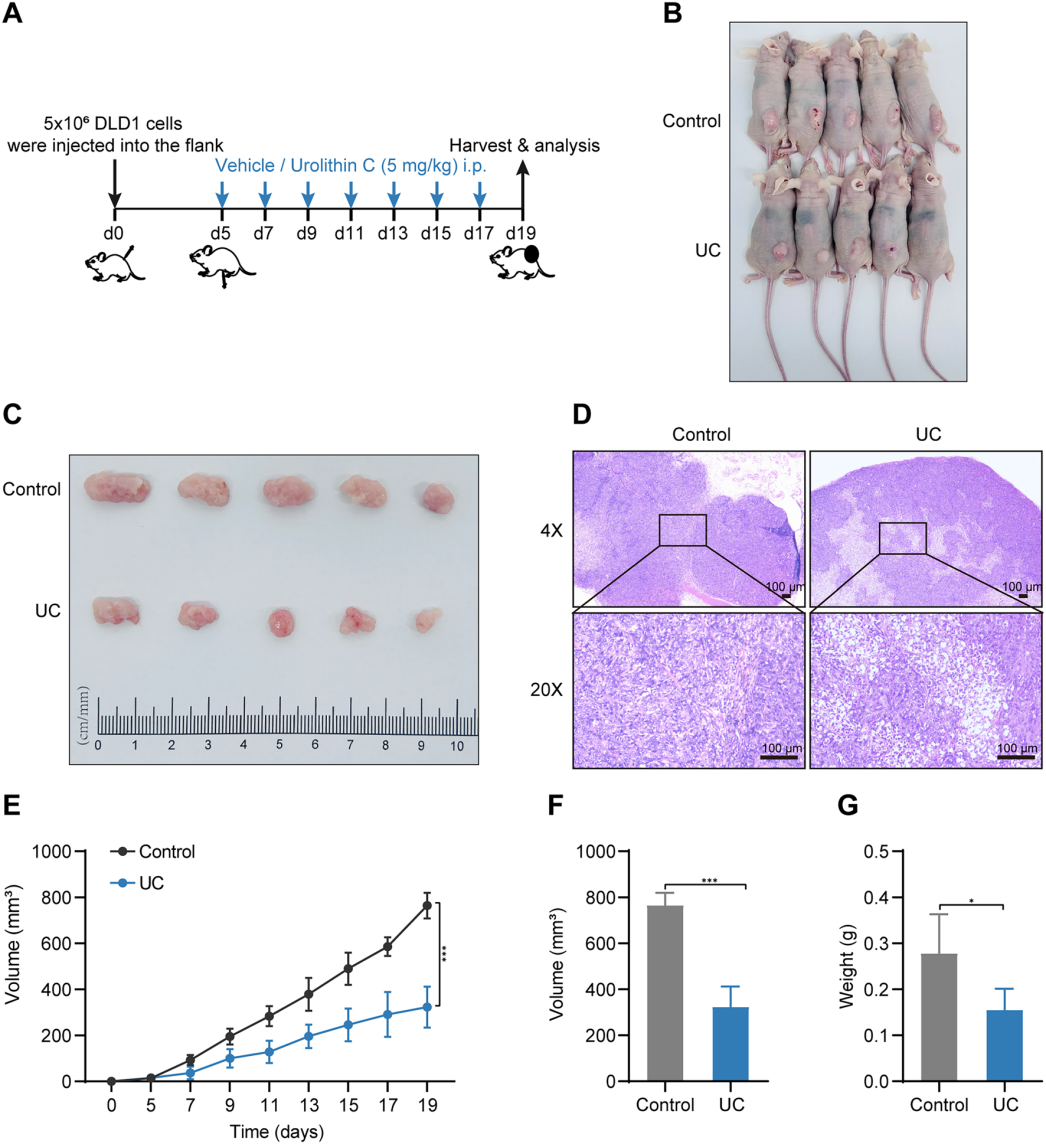
UC suppresses colorectal tumor growth in vivo*.* **A** A working model for animal experiment. **B**–**C** After 14 days of treatment, the mice were killed, and tumors were removed and photographed. **D** HE staining images were shown for every group. **E** the volume of subcutaneous tumors in the xenograft model was measured three times a week. **F**–**G** Tumor volume and weights were calculated. Data represent the means of the UC group and the control group (*n* = 5). Scale bar = 100 µm. Data were presented as mean ± SD, ns not significant, *P < 0.05, ***P < 0.001 in Student’s *t* test (**E–G)**

According to the aforementioned in vitro findings, UC was proved to be beneficial in preventing CRC cells from proliferating, inhibiting migration, and inducing apoptosis and cell cycle arrest. Since DLD1 cells had the lowest IC50 value among the three CRC cells, we chose DLD1 cells to create a *xenograft model* to investigate the antitumor capacity of UC. DMSO (control) or UC (5 mg/kg, 3 times a week) was administered by intraperitoneal injection for 14 days (Fig. 4A). Physically, the UC-treated group showed a relative reduction in tumor size compared to the control group (Fig. 4B–C). Interestingly, there was more necrosis of tumor cells in the UC-treated group on HE staining (Fig. 4D). It indicated that UC may have a key role in inhibiting tumor cell growth in vivo. As expected, tumor volume and weight were greatly reduced with the treatment of UC (Fig. 4E–G). Above all, these results strongly suggest that UC suppressed tumor growth of CRC in vivo.

### UC inhibits the expression of YBX1 in CRC cells

**Fig. 5:**
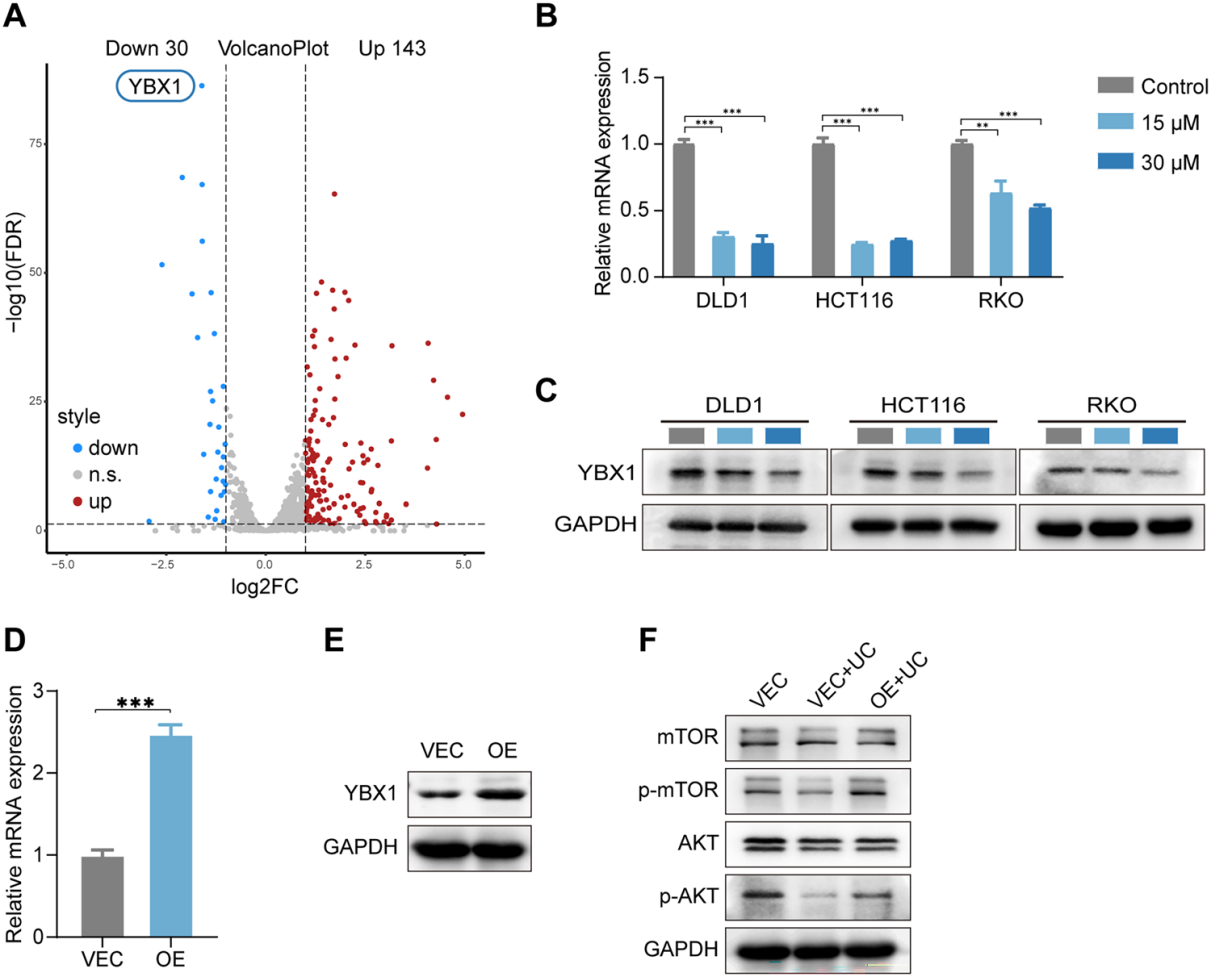
UC inhibits the expression of YBX1 in CRC cells. **A** Transcriptome analyses (RNA-seq) in DLD1 cells after treatment with UC (15 µM) for 72 h (*n* = 3). The volcano plot to visualize the number of upregulated and downregulated transcription factor genes is presented. **B** RT-qPCR assays were performed to detect the mRNA expression level of YBX1 in DLD1 cells treated with UC (15 µM and 30 μM) for 72 h (*n* = 3). **C** Western blot assays were performed to detect the protein expression level of YBX1 in DLD1 cells treated with UC (15 µM and 30 μM) for 72 h. DMSO was used as control. **D**–**E** Western blot and RT-qPCR analysis (*n* = 3) showing the overexpression efficiency of YBX1 in DLD1 cells. **F** The protein expression of the AKT/mTOR pathway in UC-treated (15 µM) DLD1.^YBX1OE^ were examined. DMSO was used as control. Data were presented as mean ± SD, **p < 0.01, ***p < 0.001 in two-way ANOVA (**B**) or in Student’s *t* test (**D)**

To further investigate the molecular mechanism of how UC acts as a potential medicine to inhibit CRC cells, RNA-seq was employed to conduct the UC (15 µM) group and DMSO (control) group. The result showed that YBX1 was the most significantly downregulated among all transcription factor genes (Fig. 5A). To further clarify the result of RNA-seq in DLD1, we examined the mRNA and protein expression of YBX1 when treated with UC (15, 30 µM). As expected, the expression of YBX1 was obviously downregulated in UC-treated CRC cells (Fig. 5B–C).

### UC suppresses YBX1-mediated AKT/mTOR pathway

YBX1, located in the nucleus, can act as an important transcription factor to regulate genes related to tumorigenesis, progression, and drug resistance [33–35]. Numerous studies show that YBX1 can promote drug resistance and cancer progression through the AKT/mTOR pathway [36]. However, how YBX1 reacts with the AKT/mTOR pathway in UC-treated CRC cells remains unclear. To elucidate the relationship between YBX1 and the AKT/mTOR signaling pathway in UC-treated CRC cells, we used YBX1 lentiviral ectopic expression vector to construct YBX1 overexpressing cell line. We used Western blot and RT-qPCR to verify the efficiency of YBX1 overexpression cell lines (Fig. 5D–E). UC suppressed the expression of the AKT/mTOR signaling pathway in CRC cells, and YBX1 overexpression greatly reversed the expression of AKT/mTOR signaling pathway when treated with UC (Fig. 5F).

**Fig. 6:**
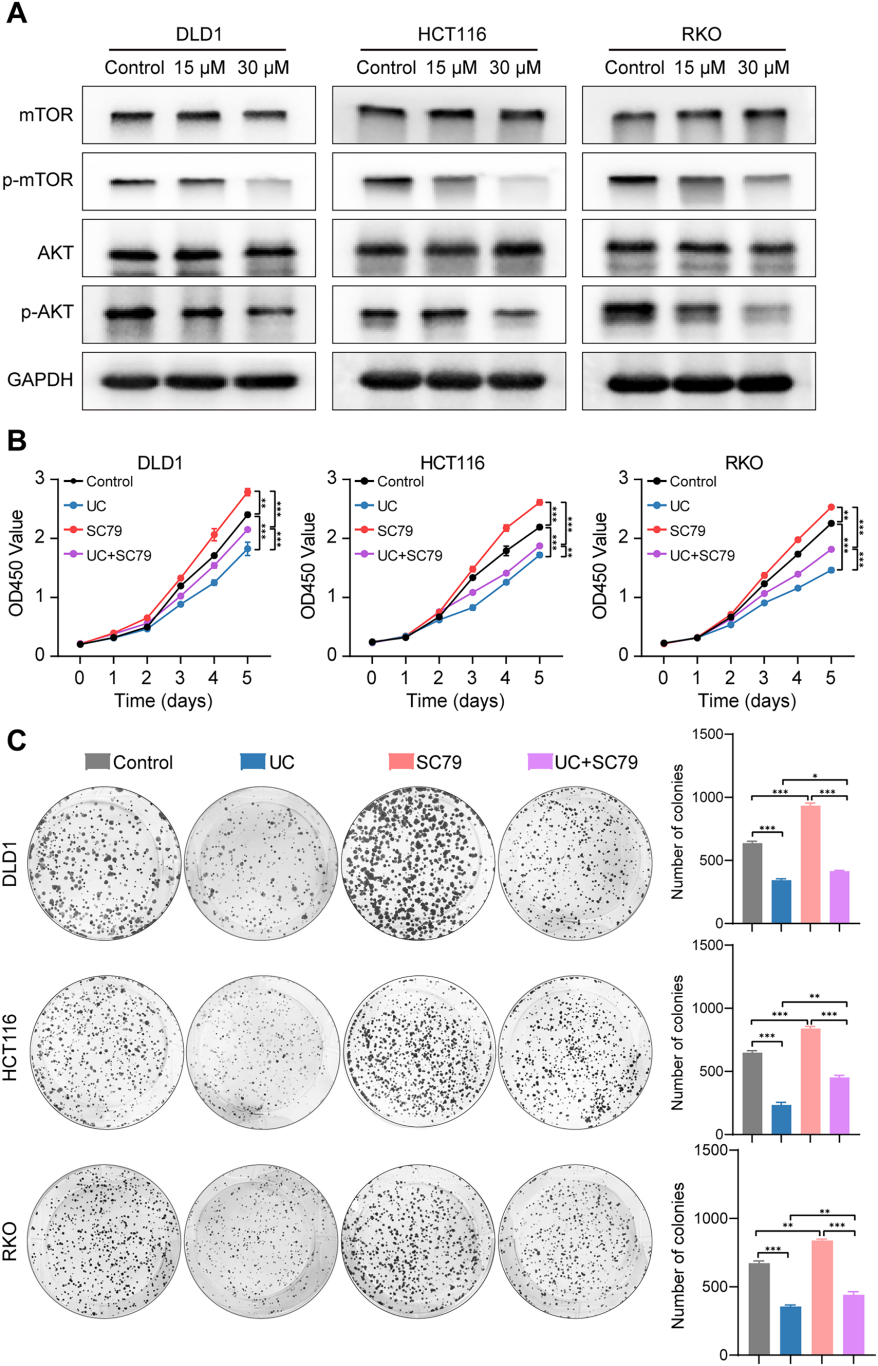
UC suppresses YBX1-mediated AKT/mTOR pathway. **A** the protein levels of phospho‐mTOR, total mTOR, phospho‐AKT, and total AKT were determined by Western blot assay analysis after treatment of UC (15 µM and 30 µM). GADPH was used as the loading controls. **B**–**C** Cell viabilities and colony formation were measured after treatment of SC79 and UC (*n* = 3). Data were presented as mean ± SD, **p < 0.01, ***p < 0.001 in Student’s *t* test (**B**–**C**)

To clarify whether UC eventually modifies YBX1 to alter the AKT/mTOR signaling pathway, the phosphorylation expression of AKT/mTOR pathway-related protein was downregulated in a UC (15, 30 µM) dose-dependent manner (Fig. 6A). Next, we added SC79 to activate the AKT signaling pathway in UC-treated CRC cells. The CCK-8 and colony formation assays results show that SC79 could reverse the inhibition of cell proliferation in UC-treated CRC cells (Fig. 6B-C). The above results indicate that UC mainly mediates the inhibitory effect on CRC via the AKT/mTOR signaling pathway.

## Discussion

In this study, we elucidated that UC inhibits the progression of CRC in vitro and in vivo and explored the deeply molecular mechanism in CRC, which suggest that UC may be a potential anticancer drug for CRC. The result showed that UC inhibits CRC cell proliferation, induces apoptosis, and arrests cell cycle in G2/M phase and inhibits the migration of CRC cells. In addition, UC suppressed tumor growth in a xenograft tumor model in vivo. Mechanically, UC inhibited the expression of YBX1 and blocked its key downstream AKT/mTOR signaling pathway. Overall, our study found that UC has antitumor effects both in vitro and in vivo, suggesting that it may have potential clinical application values.

CRC is prone to metastatic recurrence and drug resistance, which greatly reduces the effectiveness of the treatment, resulting in a lower 5 year survival rate [5]. Oxaliplatin and fluorouracil are regarded as the traditional first-line treatments for colorectal cancer and the typical second-line chemotherapy options for CRC patients include fluorouracil, folinic acid, and irinotecan (FOLFIRI) [3]. So, it may be inferred that fluorouracil, oxaliplatin, and irinotecan are traditional chemotherapeutic agents for colorectal cancer and play an important role in the treatment of colorectal cancer. However, these traditional pharmaceutical therapeutic regimens are usually accompanied by severe myelosuppression, cumulative neurosensory toxicity, and extensive hyperpigmentation [37, 38]. Thus, searching for anticancer drugs with low toxicity and minimal side effects is an urgent problem worldwide. Urolithins are metabolites of EA in the human intestinal tract, and UA and UB have been shown to have antiproliferative properties in a variety of cancers, including gastric, colorectal, lung, and pancreatic cancers [13, 14, 18, 39]. Studies have discovered that UA dose-dependently induced apoptosis, altered the expression of cell cycle-related proteins, and decreased the expression of Bcl-2 [14]. In a xenograft tumor mouse model, UA was able to prolong animal life in pancreatic cancer by inhibiting tumor development via the PI3K/AKT/mTOR pathway [18]. In addition, UB mediates the antitumor potential of CRC by arresting the G2/M phase and activating caspase 3 to induce apoptosis [22]. Besides, UB also prevents hepatocellular carcinorma cell proliferation by inactivating Wnt/β-catenin signalling [20]. Research has demonstrated that UC possesses anti-inflammatory, anticancer, antidiabetic, and antiobesity properties [25, 28, 29]. UC has been shown to have antitumor effects in prostate cancer cells, bladder cancer cells, and colorectal cancer cells [22–24, 40]. In our study, we found that UC could supress the progression of CRC via inhibiting the YBX1–AKT/mTOR signal axis. Overall, these studies imply that urolithins have a potential antitumor effect, suggesting potential clinical therapeutic values.

Our research showed that the IC50 of RKO, HCT116, and DLD1 cells were 28.81 μM, 23.06 μM, and 14.7 μM when treated with UC for 72 h. According to relevant studies, UC (20 μM) affects insulin secretion in vitro in pancreatic cells [25] and UC (20, 50 μM) has anticancer properties in colorectal cancer cells [23]. Combined with the results of our own experiments and data from the literature, we employed UC (15, 30 μM) in vitro. In our study, the IC50 of DLD1 was 14.7 μM and we used the molar concentration formula (*M* = *m*/MW*1/V) to calculate the dosage of UC (3.6 mg/kg) in vivo. UA and UB play an important role in the treatment of cancer and other disorders by oral gavage or intraperitoneal injection [16, 39, 41]. Among them, intraperitoneal injection of UA (2.5–5.0 mg/kg) reduces ischemic brain injury in mice with middle cerebral artery occlusion [42] and the potential prevention of diabetes-induced cardiac damage by intraperitoneal administration of UA and UB (2.5 mg/kg) [43]. Taken together, these investigations demonstrate the safety, rationality, and efficacy of the method of administration and dosage. Therefore, we referenced the intraperitoneal administration of UC (5.0 mg/kg) in our research.

According to the results of RNA-seq in UC-treated CRC cells, we discovered that the target transcription factor YBX1 was drastically downregulated. Previous studies have shown that the expression of YBX1 is upregulated in many cancers, including breast cancer [44], gastric cancer [45], and hepatocellular carcinoma [46]. Numerous studies have shown that YBX1 acts as an important transcription factor to regulate many biological processes which includes drug resistance and cell proliferation [47, 48] through downstream targets such as E2F, p53, and PI3K/AKT/mTOR [36]. In head and neck carcinoma, nuclear phosphorylation of YBX1 transcriptionally regulates the PI3K pathway and promotes the proliferation and invasion of head and neck cancer cells [49]. YBX1 is dependent on AKT for nuclear translocation after phosphorylation at Ser102, including ovarian cancer cells [50] and breast cancer cells [51]. Besides, YBX1 with siRNAs caused a significant reduction in mTOR protein levels in estrogen receptor-negative breast cancer cells [52]. In glioma, YBX1 may act as an important activator of the mTOR signaling pathway and mediate the YBX1/CCT4/mLST8/mTOR axis to promote the growth of glioblastomas [53]. In our study, SC79 reverses the inhibition of cell proliferation in UC-treated CRC cells via the YBX1-mediated AKT/mTOR signaling pathway. Taken together, YBX1 has an important role in the AKT/mTOR pathway. As expected, we demonstrated that UC inhibits CRC progression by inhibiting the AKT/mTOR pathway via downregulating the expression of YBX1.

**Fig. 7:**
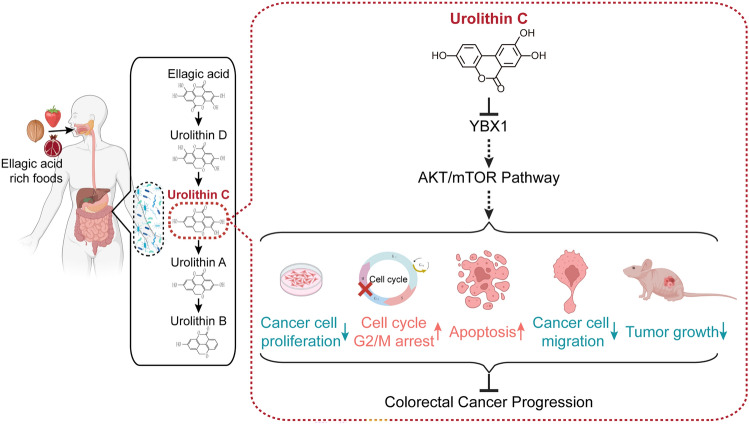
A proposed working model of the mechanism. UC exerts antitumor effects by inhibiting the AKT/mTOR pathway and thereby suppressing CRC progression

Regarding the security of urolithin supplementation, previous clinical investigation showed that daily supplementation with 1000 mg of UA for 4 months was safe and could enhance mitochondrial function and muscular endurance in healthy elderly people [54, 55]. The above clinical trials suggested that urolithins have potential clinical applications. In this study, UC as a natural compound was found to have antitumor effect in CRC. However, it should be emphasized that our study is still in a preliminary stage and more experimental investigations and clinical trials are required to validate the antitumor activity of UC as well as to investigate its potential for application in the clinic and future safety. In conclusion, this study demonstrated that UC supresses the progression of CRC via inhibiting the YBX1–AKT/mTOR signal axis (Fig. 7), which provided functional evidences for the application of UC in the treatment of CRC in clinical practice.

### Supplementary Information

Below is the link to the electronic supplementary material.

Supplementary file1 (DOCX 591 KB)

## Data Availability

All data have been shown in the manuscript.

## Abbreviations

CCK-8: Cell Counting Kit-8
CRC: Colorectal cancer
DMSO: Dimethyl sulfoxide
EA: Ellagic acid
FBS: Fetal bovine serum
FCM: Flow cytometry
HE: Hematoxylin–eosin staining
IC50: 50% Inhibitory concentration
PBS: Phosphate-buffered saline
RNA-seq: RNA sequencing
RT-qPCR: Quantitative reverse transcription polymerase chain reaction
UA: Urolithin A
UB: Urolithin B
UC: Urolithin C
WB: Western blot
YBX1: Y-box binding protein 1
