# Supplementary figures and images for: Urolithin C suppresses colorectal cancer progression via the AKT/mTOR pathway
Source: J Nat Med. 2024 Jun 7;78(4):887–900. doi: 10.1007/s11418-024-01821-2 (PMC11364574; doi:10.1007/s11418-024-01821-2)

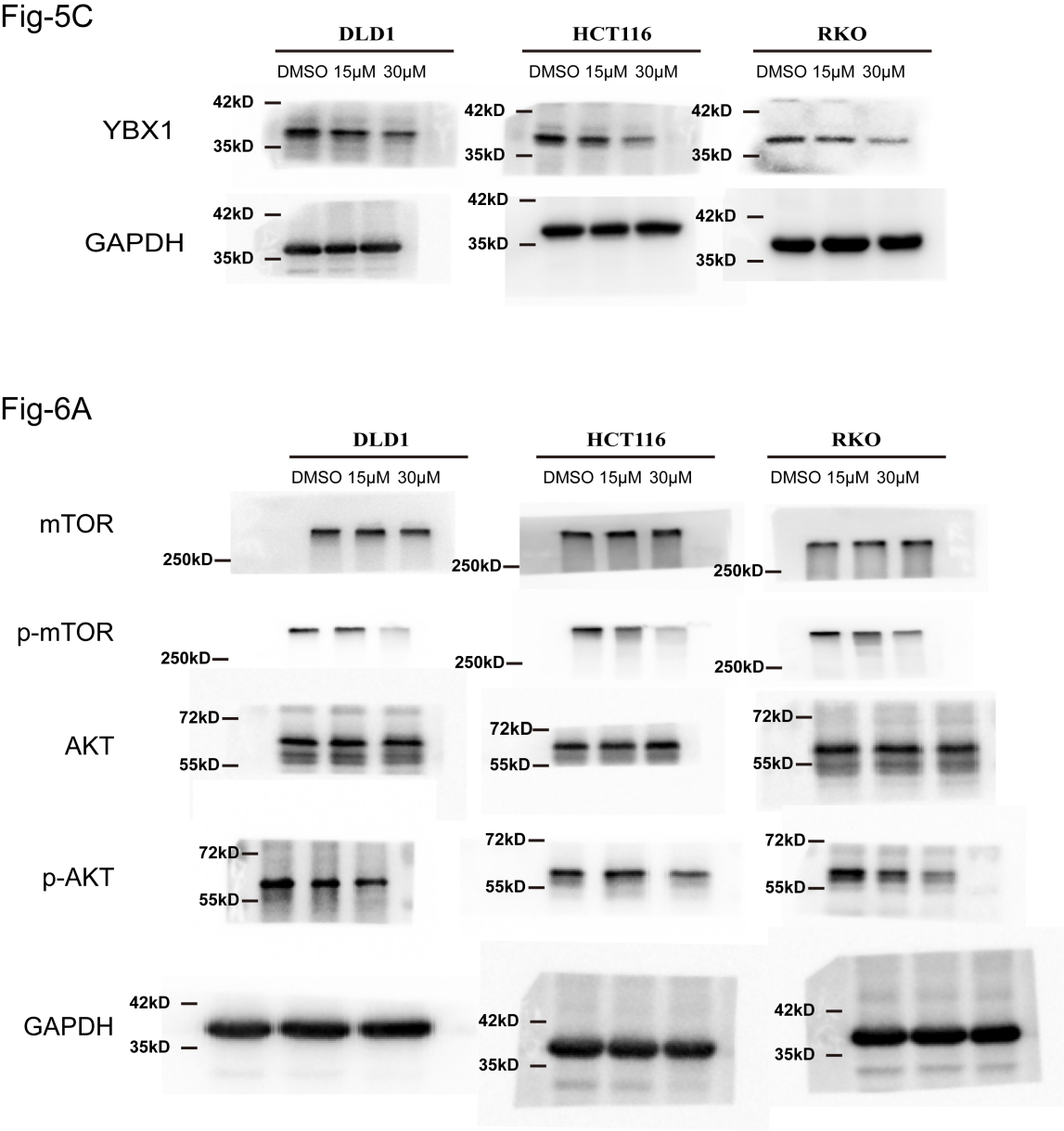

Supplement: Supplementary file 1 — Supplementary file1 (DOCX 591 KB) [file 11418_2024_1821_MOESM1_ESM.docx]
